# Translation and Cultural Adaptation of the PedsQL Neurofibromatosis Module, Version 3.0, Into Brazilian Portuguese

**DOI:** 10.7759/cureus.103088

**Published:** 2026-02-06

**Authors:** Marília Oliveira Machado, Fernanda T de Lima, Amanda S do Nascimento, Nasjla S Saba, Andrea M Capellano, Eliana Monteiro Caran

**Affiliations:** 1 Pediatric Oncology, GRAACC, Universidade Federal de São Paulo (UNIFESP), São Paulo, BRA; 2 Genetics, GRAACC, Universidade Federal de São Paulo (UNIFESP), São Paulo, BRA

**Keywords:** child and adolescent, neurofibromatosis type 1, peds ql, quality of life (qol), young adults

## Abstract

Introduction: Neurofibromatosis type 1 (NF1) is a prevalent genetic disorder for which no pharmacological treatment is currently available. It affects approximately 1 in 3,000 live births, with an estimated 80,000 individuals living with the condition in Brazil. Benign tumors, such as plexiform neurofibromas, can substantially impair both physical function and appearance. Additionally, patients can present behavioral and cognitive challenges, including language impairments, learning disabilities, hyperactivity, autism spectrum symptoms, and depression. The quality of life of affected individuals is largely influenced by clinical, psychological, and social factors. Quality-of-life assessment cannot rely solely on clinical observation; it requires direct input from patients and their families. Validated instruments, such as the PedsQL NF1 Module, are essential to objectively capture these perspectives.

Objective: To translate and culturally adapt the *Scaling and Scoring of the Pediatric Quality of Life Inventory *(PedsQL) Neurofibromatosis Module into Brazilian Portuguese for children, adolescents, and young adults with NF1 (ages 5-25), as well as their parents or caregivers.

Methods: The study followed four phases: (1) translation into Portuguese; (2) back-translation into English; (3) expert review by physicians and nurses from multiple regions of Brazil; and (4) pilot testing with NF1 patients. Expert evaluation included the Content Validity Index (CVI) and Kappa coefficient to assess agreement and reliability.

Results: The PedsQL NF1 Module was successfully translated and culturally adapted for Brazilian patients aged 5-25 years. The results showed satisfactory CVI (0.75) and Kappa (0.6) values. Minor adjustments were made to the caregiver version to improve comprehension, e.g., replacing *fear* with *is afraid* for better contextual meaning. The pilot study included 42 patients and 46 caregivers. Five patients with behavioral conditions (autism spectrum disorder or hyperactivity) were unable to complete the questionnaire.

Conclusions: The translation, cultural adaptation, and pre-testing of the PedsQL NF1 Module were successfully completed. The instrument demonstrated acceptable content validity and inter-rater agreement, supporting its applicability for Brazilian patients and caregivers.

## Introduction

Neurofibromatosis type 1 (NF1) is an autosomal dominant genetic disorder characterized by complete penetrance and variable expression. It affects approximately 1 in 3,000 live births, with an estimated 80,000 individuals living with the condition in Brazil [1,2]. NF1 results from the functional loss of one allele of the NF1 gene, located on chromosome 17q11.2. This gene encodes neurofibromin, a protein that inactivates Ras protein kinase, a key regulator of cell growth and division. Impaired neurofibromin function leads to Ras hyperactivation, contributing to the development of both benign and malignant tumors [1,3].

NF1 presents with a wide range of phenotypic variations, from mild and asymptomatic to severe and disabling manifestations. These symptoms may be progressive, with new clinical features emerging over time [4,5]. Diagnosis is primarily clinical, based on criteria established by the National Institutes of Health (NIH) in 1987, and subsequently revised in 1997 and 2021 [6]. Benign tumors such as plexiform neurofibromas can substantially impair both physical function and appearance. Additionally, behavioral and cognitive challenges, including language impairments, learning disabilities, hyperactivity, autism spectrum symptoms, and depression, further complicate management and negatively impact quality of life [7-9].

Currently, there is no curative treatment for NF1. Therefore, patients’ quality of life depends on comprehensive management of the disease’s clinical, psychological, and social aspects [10,11].

This study aimed to translate and culturally adapt the Scaling and Scoring of the Pediatric Quality of Life Inventory Neurofibromatosis Module, Version 3.0 (PedsQL 3.0), into Brazilian Portuguese for use in children, adolescents, and young adults with NF1. Once validated, this tool will support the identification of the specific needs of this population and inform the development of more effective and inclusive strategies [12,13].

Health-related quality of life (HRQoL) assessment tools are widely used in healthcare, yet they are often developed in English and applied to culturally distinct populations [14]. To ensure that the conceptual integrity of such instruments is preserved, it is essential to undertake not only accurate translation but also careful cultural adaptation [15]. Adapting a validated instrument is typically more cost-effective than developing a new one, and it facilitates the use of standardized tools for cross-cultural comparisons and scientific collaboration [16-18].

This research was initially submitted as a master's thesis to the Universidade Federal de São Paulo (UNIFESP) on February 17, 2023 (https://repositorio.unifesp.br/items/be3fd4f3-07ec-4509-8a1a-635aab5d88fc). The findings were also presented as an abstract at the 2023 Brazilian Society of Pediatric Oncology Congress (SOBOPE) and later as a poster at the International Society of Pediatric Oncology Congress (SIOP).

## Materials and methods

This study was approved by the Research Ethics Committee of the Federal University of São Paulo (UNIFESP) under protocol number 4.332.450. Authorization to use the original English-language questionnaire was obtained from the MAPI Research Trust, which manages permissions for PedsQL instruments.

**Figure 1:**
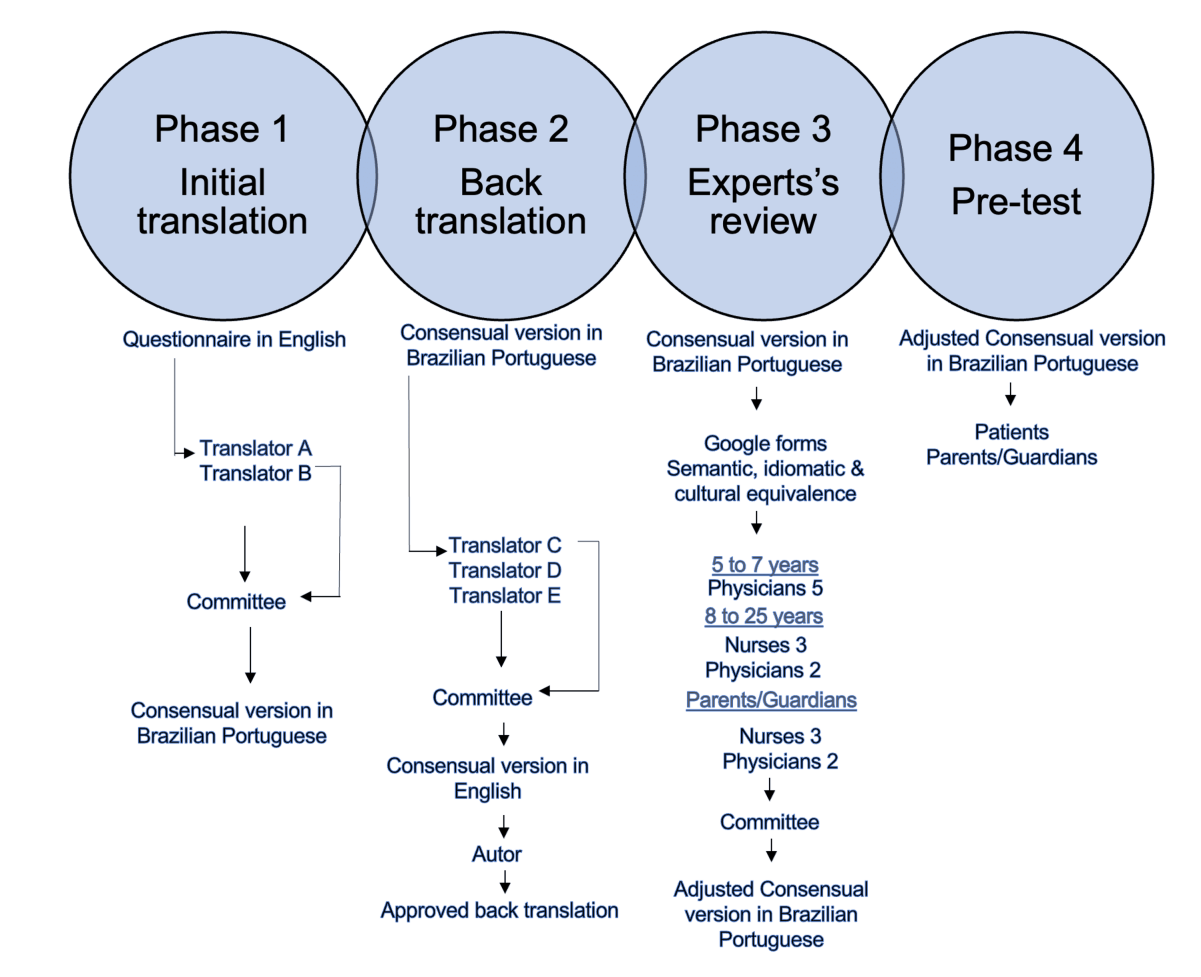
Translation and adaptation flowchart. Image credit: Fernanda T. de Lima. Created using PowerPoint.

The translation and cultural adaptation process followed the methodology proposed by Guillemin et al. (1993), which is widely cited in the literature for HRQoL instruments (Figure 1) [19-23]. This methodology was selected due to its proven reproducibility and successful application in previous studies [14,22].

The study was conducted between December 2020 and January 2022 at a pediatric oncology hospital, where approximately 70% of patients received care through the Brazilian Unified Health System (SUS). A demographic and clinical data form was completed for each participant, including information on education level and family history of NF1.

The PedsQL 3.0 NF1 Module is a multidimensional instrument specifically developed for NF1. It includes 18 scales across various domains and is available in eight versions tailored to four age groups (5-7, 8-12, 13-18, and 19-25 years), each with corresponding caregiver versions [12]. The study included patients aged 5 to 25 years and their parents or guardians, who completed the age-appropriate version of the questionnaire. A partial reproduction of the English version for children aged 8 to 12 years is included in the Appendix**.**

Responses are recorded on a five-point Likert scale: 0 = “never,” 1 = “almost never,” 2 = “sometimes,” 3 = “often,” and 4 = “almost always.” For the 5-7-year-old group, a three-face visual scale was used to facilitate understanding, with expressions corresponding to “never” (0), “sometimes” (2), and “always” (3). Total scores were transformed into a 0-100 scale, with higher scores indicating better perceived quality of life.

Translation and adaptation process

Phase 1: Initial Translation

Two independent bilingual translators (non-healthcare professionals) translated the original questionnaire into Brazilian Portuguese. A local committee composed of a nurse and a pediatric oncologist reviewed both versions and synthesized a consensus version, ensuring semantic and conceptual equivalence.

Phase 2: Back-translation

Three native English-speaking translators with proficiency in Portuguese, and no prior knowledge of the study, back-translated the consensus version. The original developers of the instrument reviewed the back-translation and approved it without reservations [20].

Phase 3: Expert Review

Using a Google Forms platform, 15 healthcare professionals from various Brazilian regions evaluated the original, translated, and back-translated versions. The evaluation followed the recommendations of Nóbrega and Gutiérrez (2000) [21], focusing on regional linguistic appropriateness and conceptual clarity.

Phase 4: Pre-test With Patients and Caregivers

The final Portuguese version was administered during routine outpatient visits by trained nurses. Table 1 displays the age distribution of participating patients and caregivers.

**Table 1:** Distribution of patients and caregivers by age group. PedsQL NF1 Module: Translated Versions by Age Group. Credit: Marília Oliveira Machado. Created using Microsoft Excel. PedsQL NF1, Scaling and Scoring of the Pediatric Quality of Life Inventory Neurofibromatosis Type 1 Module

PedsQL questionnaire NF1 translation version	5-7 years old	8-12 years old	13-18 years old	19-25 years old	Total
Patients with NF1	10	18	8	6	42
Parents/guardians of patients with NF1	13	20	7	6	46

Statistical analysis

Descriptive statistics were used to summarize demographic and clinical data. Continuous variables were expressed as means and medians, while categorical variables were presented as frequencies and percentages. The Content Validity Index (CVI) and Kappa coefficient were used to assess inter-rater agreement, with CVI > 0.75 and Kappa > 0.62 considered satisfactory. All analyses were conducted using R software, version 4.1.2 (R Core Team, R Foundation for Statistical Computing, Vienna, Austria).

## Results

During the translation phase, no significant discrepancies were observed between the two initial Portuguese versions produced in Phase 1. A final consensus version was developed by the nurse and pediatric oncologist, incorporating the most appropriate elements from both translations. At the end of Phase 2, the back-translated English version, prepared by three independent translators, showed no inconsistencies with the original instrument. The authors of the questionnaire reviewed and approved the back-translation without reservations [20].

**Figure 2:**
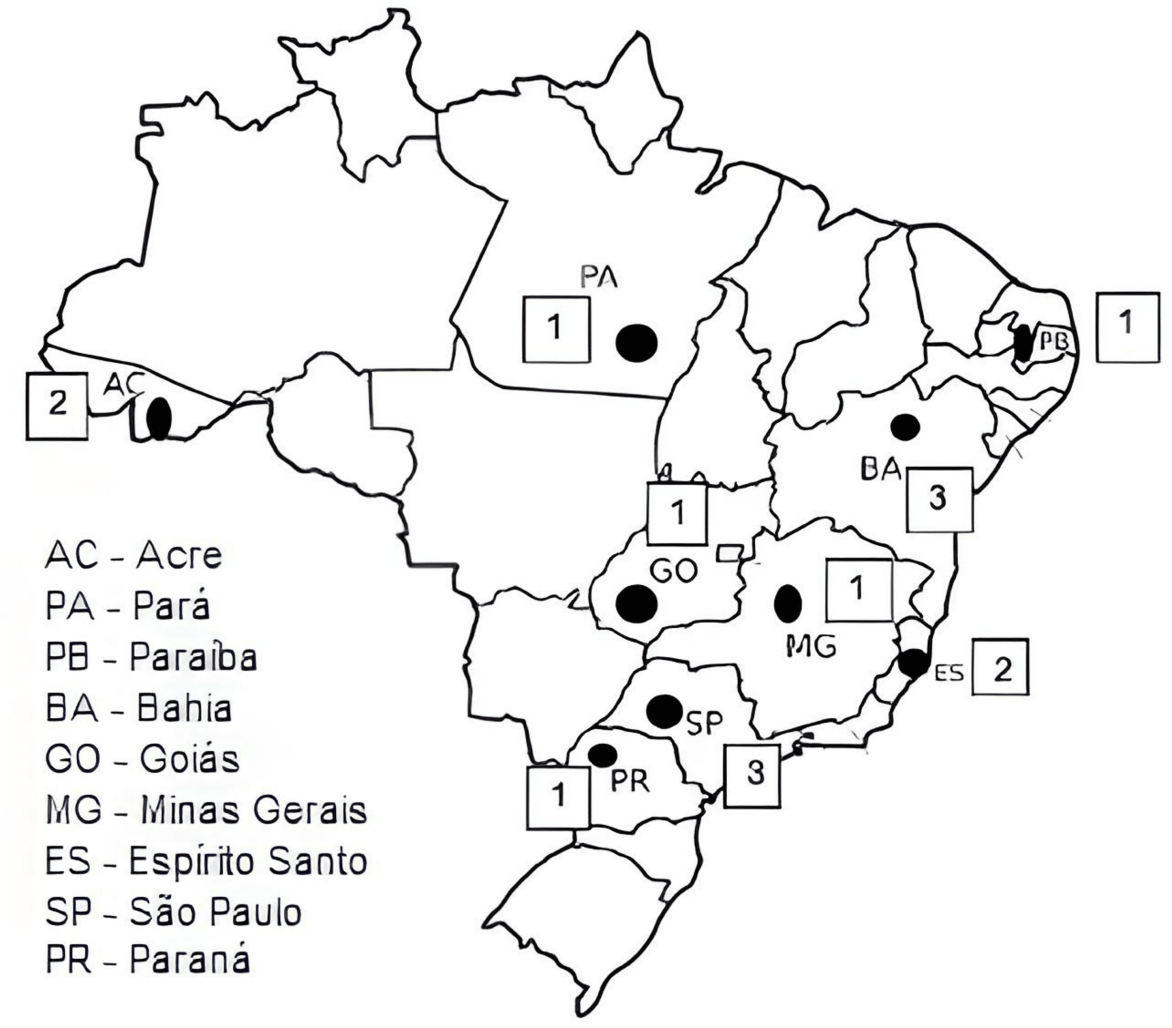
Map of Brazil showing the states and number of professionals who participated in Phase 3. Image credit: Marília Oliveira Machado. Created using PowerPoint.

In Phase 3, 15 healthcare professionals from different Brazilian states participated in the expert review process. Using a Likert scale, they evaluated the semantic and conceptual equivalence between the Portuguese, back-translated, and original versions. This evaluation took into account Brazil’s regional linguistic and cultural diversity (Figure 2).

**Figure 3:**
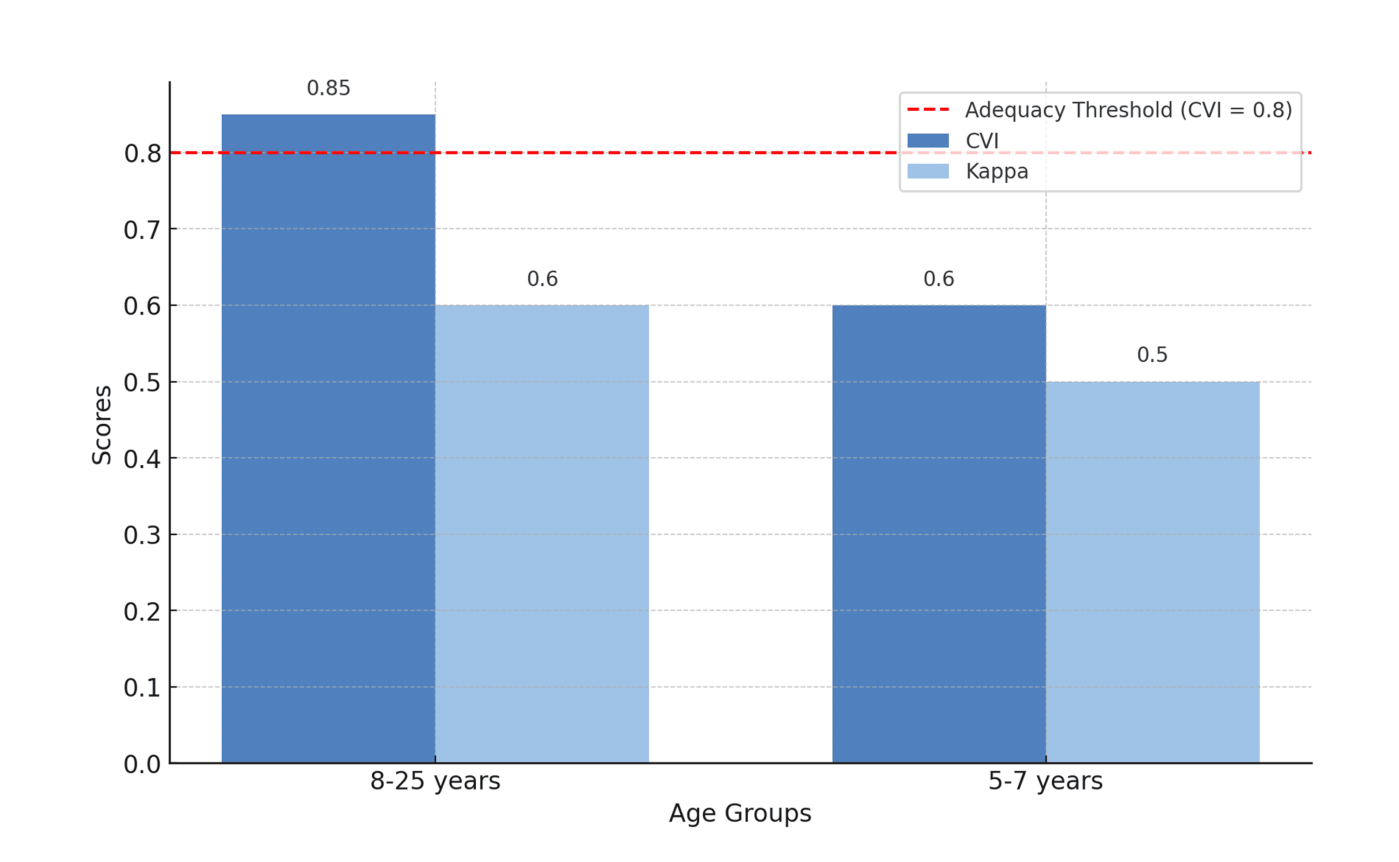
CVI and Kappa scores by age group. Figure credits: Marília Oliveira Machado. Created using PowerPoint. CVI, Content Validity Index

According to the expert panel, the translated and back-translated questionnaires for patients aged 8 to 25 years demonstrated strong agreement, with CVI values above 0.80 and Kappa coefficients exceeding 0.6. However, for the 5-7-year-old age group, the item “Do you get an upset stomach?” in the stomach discomfort domain received a CVI of 0.6 and a Kappa of 0.5, indicating partial disagreement (Figure 3). Evaluators suggested replacing “stomachache” with “discomfort in your stomach” to enhance clarity (Table 2).

**Table 2:** Concordance between patient and caregiver responses was assessed using the chi-square test. Table credit: Marília Oliveira Machado. Created using Microsoft Excel.

Variable	Interpretation	*P*-value
Pain intensity	No concordance (significant difference)	<0.05
Pain frequency	Concordance (no significant difference)	0.487
Paying attention	Concordance (no significant difference)	0.250

In the caregiver version, the item “Skin bothers him/her more when it is hot” also raised concerns. The translation was considered vague, receiving a CVI of 0.4 and a Kappa of 0.25. Eight other caregiver items-particularly those related to pain and concerns-received CVI scores of 0.6 and Kappa values of 0.5. For instance, in the pain domain, the item “Feeling pain or hurt in his/her stomach” was translated as “He/she feels stomach pain,” which was considered insufficient to convey the original nuance.

Additional feedback included replacing the word “fears” with “is afraid” in the concerns domain, as in “is afraid of not being liked by others.” Similarly, in the stomach discomfort domain, expressions such as “burning sensation” were suggested to be replaced with “discomfort” to avoid misinterpretation.

**Figure 4:**
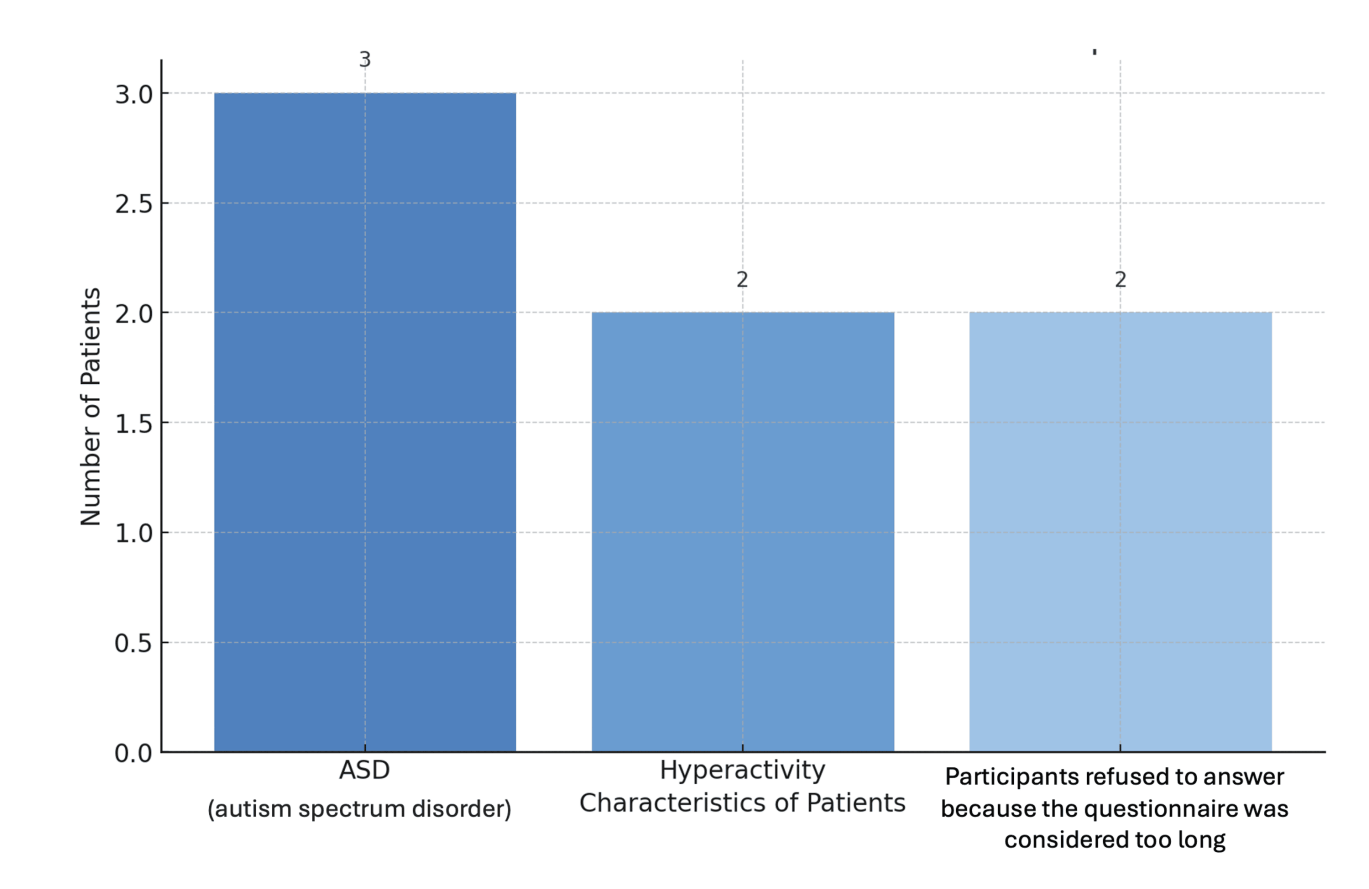
Characteristics of patients who did not complete the questionnaire. Image credit: Marília Oliveira Machado. Created using PowerPoint.

**Figure 5:**
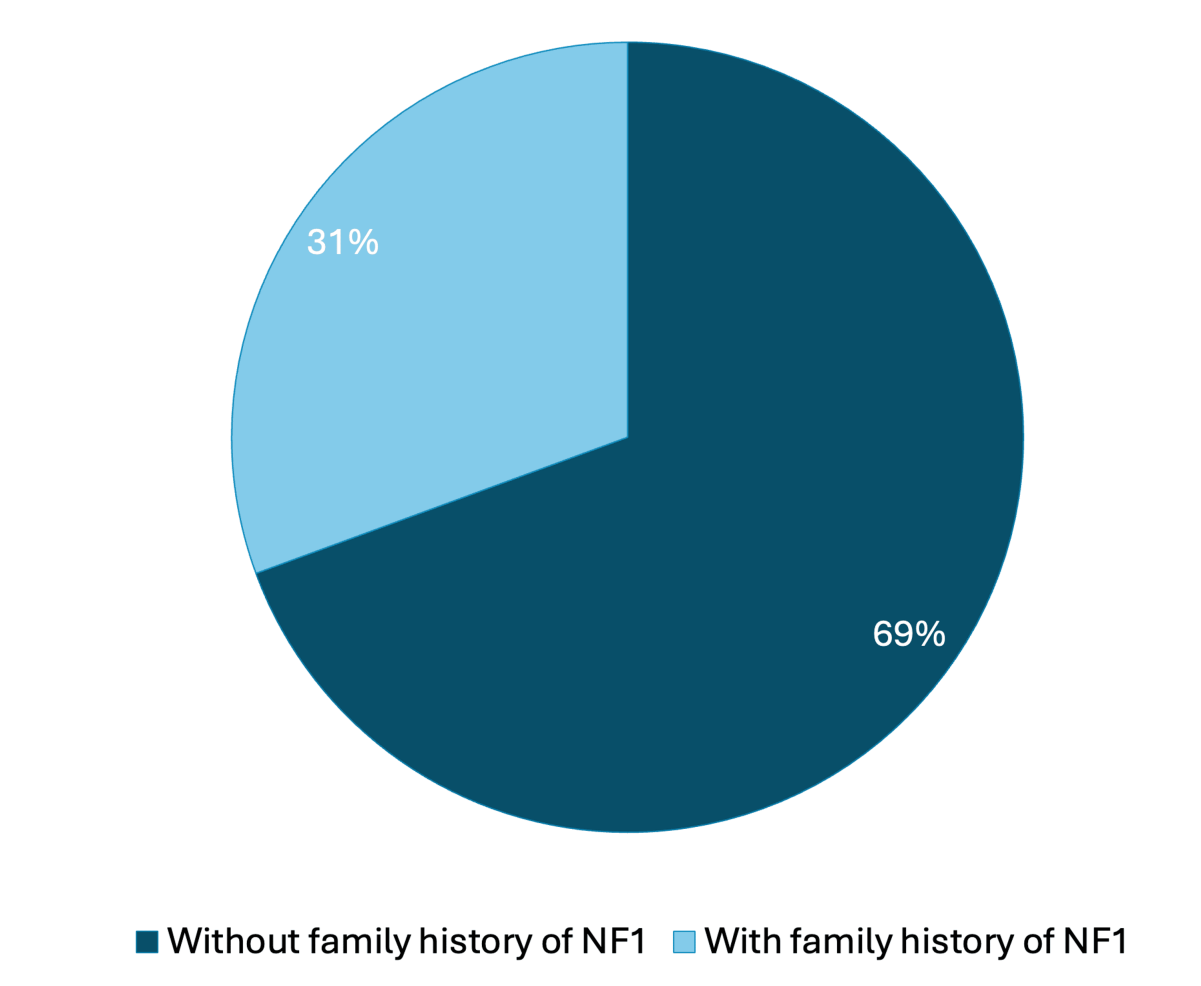
Family history of NF1 among patients. Image credits: Marília Oliveira Machado. Created using PowerPoint. NF1, neurofibromatosis type 1

In total, 49 patients and caregivers were recruited, with 42 patients and 46 caregivers completing the questionnaire. Non-completion occurred in seven cases: three with autism spectrum disorder, two with hyperactivity, and two who found the questionnaire too lengthy (Figure 4). The patients ranged in age from 5 to 25 years, with a mean age of 10.5 and a median of 10 years. Most participants were female (28/49, 57.1%), and 34/49 (70.8%) reported no family history of NF1 (Figure 5).

Two caregivers declined to participate due to limited contact with the patient, and one refused to complete the form.

**Figure 6:**
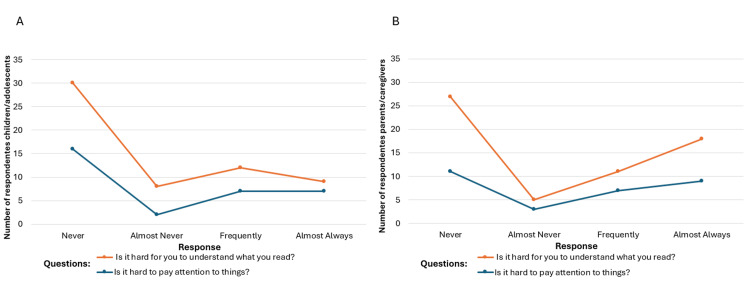
Cognitive function: (A) child/adolescent self-reports; (B) parent/caregiver proxy reports. Image credit: Marília Oliveira Machado. Created using Excel.

**Figure 7:**
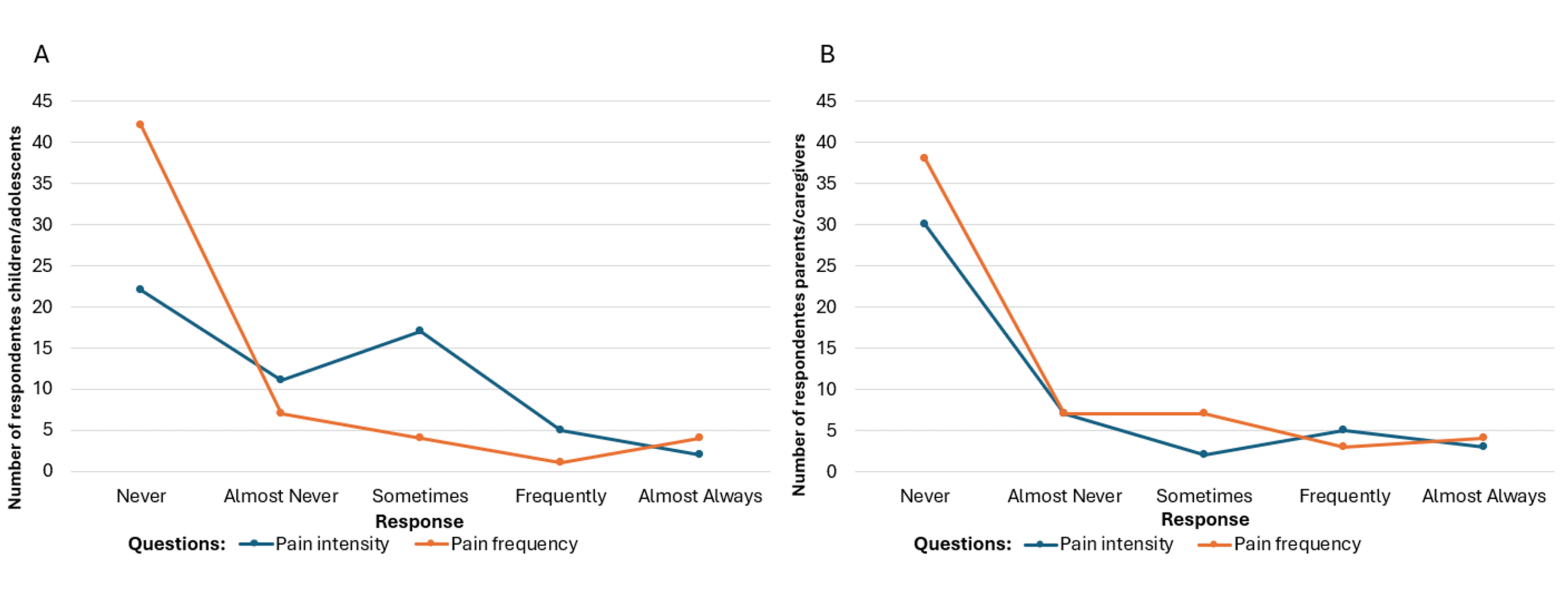
(A) Pain intensity reported by children/adolescents and caregivers and (B) pain frequency reported by children/adolescents and caregivers. Image credit: Marília Oliveira Machado. Created using Excel.

After applying the instrument, graphical analyses were created to visualize perspectives from both patients and caregivers. These included figures on cognitive function (Figure 6) and pain intensity/frequency (Figure 7).

To assess agreement between patients and caregivers, chi-square tests were performed on four variables: pain intensity, pain frequency, attention, and reading comprehension. Significant disagreement was found in pain intensity and reading comprehension (*P* < 0.05), while agreement was observed for pain frequency (*P* = 0.487) and attention (*P* = 0.250) (Table 2).

**Figure 8:**
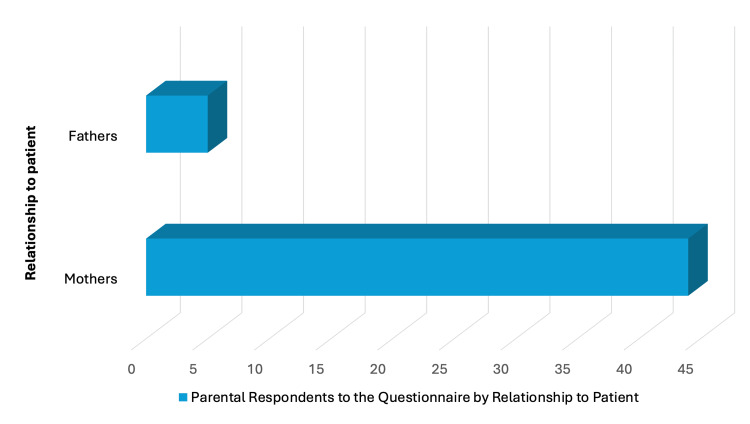
Relationship of parental respondents to the patient. Image credit: Marília Oliveira Machado. Created using PowerPoint.

**Figure 9:**
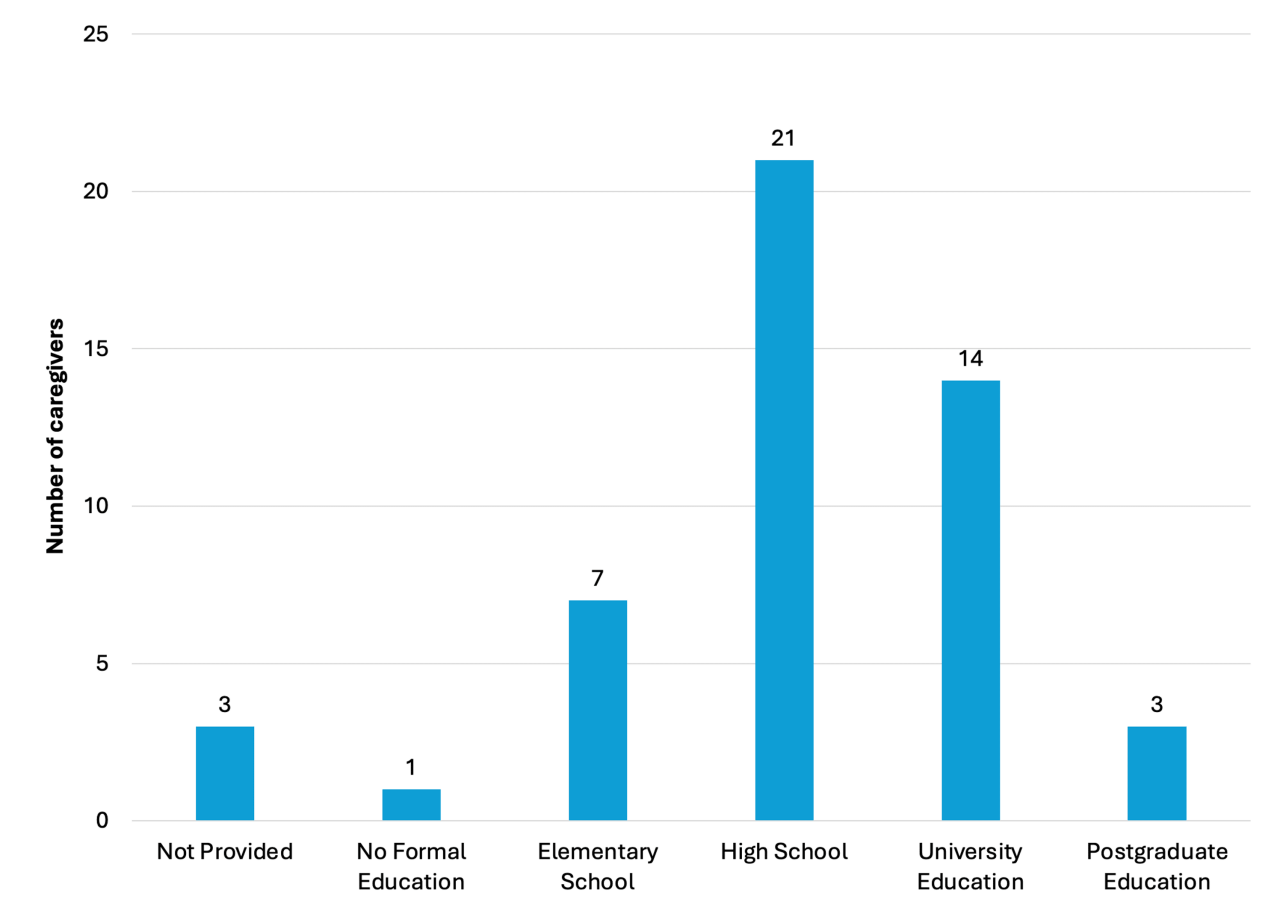
Caregivers' educational level. Image credit: Marília Oliveira Machado. Created using PowerPoint.

Regarding caregiver demographics, ages ranged from 28 to 54 years (mean = 40.6; median = 42). Most respondents were mothers (44/49, 89.7%; Figure 8).​​​​​​​ Educational attainment included postgraduate education (3/49, 6.1%), university degree (14/49, 28.5%), high school (22/49, 42.8%), elementary school (7/49, 14.2%), no formal education (1/49, 2.0%), and not disclosed (3/49, 6.1%) (Figure 9).​​​​​​​

## Discussion

Given the complexity of NF1, patients often experience a wide range of physical, emotional, and social challenges [24,25]. Multidisciplinary teams must recognize that quality-of-life assessment cannot rely solely on clinical observation; it requires direct input from patients and their families. Validated instruments, such as the PedsQL NF1 Module, are essential for objectively capturing these perspectives.

In clinical practice, patients with NF1 exhibit marked variability in disease expression, which may be progressive with advancing age. Therefore, the availability of a disease-specific instrument that incorporates the perspectives and concerns of individuals with NF1 and their families is essential for understanding the impact of the condition across different age groups, cultures, religions, and socioeconomic backgrounds. Identifying the aspects of the disease that are most relevant to patients and their families enables the individualization and optimization of care strategies, contributing to the delivery of comprehensive, patient-centered care for individuals with NF1 [26-30].

Patients with NF1 present frequent and heterogeneous alterations of the central nervous system (CNS), encompassing structural, functional, and emotional aspects. Structural abnormalities include macrocephaly, hydrocephalus, aqueductal stenosis, and vasculopathies, while functional impairments involve epilepsy, attention deficits, cognitive dysfunction, and autism spectrum disorder [3,5,6,26].

Magnetic resonance imaging (MRI) studies of the CNS have investigated the correlation between white matter abnormalities and functional impairment, particularly visuospatial processing. In addition, patients with NF1 exhibit reduced levels of the neurotransmitter gamma-aminobutyric acid (GABA) in the occipital and frontal lobes [27]. However, cognitive dysfunctions in these patients result from multiple pathophysiological mechanisms that are not yet fully understood [26,28].

Emotional factors also contribute significantly to behavioral changes. According to individuals with NF1, factors associated with painful emotional and social experiences include altered physical appearance, fear of transmitting the disease to offspring, learning difficulties, limitations in performing daily activities, chronic pain, rejection, social isolation, and the perception of insufficient social support. Therefore, psychological and psychopedagogical support should be an integral part of the multidisciplinary follow-up of these patients, enabling them to develop coping skills to better manage the suffering they experience [10,11].

The PedsQL instrument was originally developed by Varni and colleagues, and the Neurofibromatosis Module 3.0 was described and published in a study by Nutakki et al. [12]. The PedsQL 3.0 NF1 Module addresses core symptoms commonly seen in NF1, including pain, itching, motor and cognitive impairments, communication difficulties, and gastrointestinal issues [7,8]. 

This instrument is particularly useful for families with no prior knowledge or experience with the disease. In our study, the majority of patients (70%) reported no family history of NF1, which differs from the literature, where approximately 50% of cases do not present a familial history of the disease [1,2]. This discrepancy likely reflects underdiagnosis of NF1 in adults, variability in clinical expression, or limited disease awareness among family members, factors that are particularly common in developing countries.

Patients without a known family history of NF1 would benefit substantially from the validation of this questionnaire, as it provides an opportunity to expand knowledge about the manifestations of the disease. However, the use of international questionnaires in Brazil requires careful validation to ensure internal consistency, appropriate item correlation, and reproducibility of results after adaptation to different social, economic, and cultural contexts.

During the adaptation process, some caregiver items required linguistic adjustments. For example, the item “Skin bothers him/her more when it is hot” was considered unclear due to ambiguity in the term “hot.” Reviewers proposed “Does his/her skin get more irritated in the heat?” to specify environmental temperature.

In the pain domain, the original English version differentiates between “hurt” and “pain,” whereas the Portuguese version used only “pain.” While this did not hinder comprehension, it may have affected the reviewers’ perception of semantic accuracy (Table 3).

**Table 3:** Main modifications and terminological adjustments for cross-cultural adaptation in Brazil. Table credits: Marília Oliveira Machado. Created using Microsoft Word.

Original version	Reviewer considerations
My skin feels like pins and needles.	In the North/Northeast regions: “My skin feels like it is prickling.”
Does your/his/her skin tingle?	Suggested phrasing: “He/she feels a tingling sensation on the skin,” to avoid ambiguity.
His/her skin feels like pins and needles.	“He feels like he has pins and needles on his skin.”
His/her skin feels like it is burning.	“Does he/she feel a burning sensation on the skin?”
It is hard for me to remember what I was just thinking.	Suggested equivalent: “My memory is weak.”
It is hard for me to do math.	Preferable: “To perform mathematical calculations.”
Do you forget schoolwork that you need to do?	Suggested terms: “task,” “lesson,” or “homework.”
I feel sick to my stomach.	Suggestion: “I feel nauseous” (add “nausea or the urge to vomit” in parentheses).
I have to push hard to have a bowel movement.	Suggest including in parentheses: “to defecate” or “to poop.”
Do you get an upset stomach?	Suggested translation: “Do you feel discomfort in your stomach?”
Difficulty moving around when he/she has pain	Suggested revision: “Do you have difficulty moving when in pain?”
Difficulty sleeping without pain medication	Replace “analgesics” with “pain medication” for better understanding.
Is it hard to have fun when you have pain?	Replace “have fun” with “play” for easier comprehension.
Is it hard for you to tie your shoes?	Add “shoelaces” for clarity.
Do you not like other people to see your bumps (fibromas or tumors)?	Suggested: “swellings = nodules/lumps.”

Overall, the translation process was strengthened by input from healthcare professionals across Brazil, ensuring culturally sensitive adaptations. Terms such as *pins and needles* were translated using regionally familiar expressions like *prickling*. Despite Brazil’s cultural heterogeneity, consensus was reached for most items.

Factors such as education, socioeconomic status, and disease severity are known to influence HRQoL outcomes [4,8,12,25]. These variables should be addressed in future validation studies to enhance instrument generalizability.

Although most items surpassed the acceptable CVI and Kappa thresholds, the observed discordance between patients and caregivers, especially in pain intensity and reading comprehension, highlights differing perspectives. These findings reinforce the value of including both respondent types in NF1 research.

Limitations

One major limitation was the small sample size, which, although acceptable for translation and cultural adaptation, is insufficient for psychometric validation. Contributing factors included the rarity of NF1 and logistical challenges imposed by the COVID-19 pandemic.

Another notable challenge was the high prevalence of behavioral disorders among participants. As highlighted in a previous study, approximately 38% of children with NF1 met criteria for attention-deficit/hyperactivity disorder (ADHD) [25]. Thus, the presence of neurobehavioral comorbidities represents not only a practical limitation but also reflects the real-world clinical complexity of NF1. These comorbidities likely affected the feasibility of questionnaire administration in certain subgroups, and we believe this challenge to be common in the application of the instrument across different cultures and languages. Future research should explore strategies to optimize the questionnaire, including simpler formats and adaptations tailored for neurodivergent populations.

## Conclusions

The translation, cultural adaptation, and pre-testing of the PedsQL 3.0 NF1 Module for Brazilian patients aged 5 to 25 years and their caregivers were successfully conducted. Most items achieved satisfactory semantic and conceptual equivalence, as supported by expert review.

Some items, particularly those related to stomach discomfort, dermatological symptoms, and appearance-related concerns in the caregiver version, required targeted linguistic refinement.

The presence of behavioral disorders, such as hyperactivity and ASD, posed challenges during administration. Additionally, the analysis revealed significant differences between patient and caregiver responses in certain domains, underscoring the importance of incorporating both perspectives when assessing quality of life in NF1.

Future validation studies, especially those conducted across multiple Brazilian regions, will be crucial to confirm the instrument’s reliability and ensure it reflects the lived experience of NF1. These efforts will help tailor interventions and inform public health strategies.

In conclusion, the Brazilian Portuguese version of the PedsQL 3.0 NF1 Module demonstrates promise as a culturally appropriate tool for assessing quality of life in children, adolescents, and young adults with NF1, provided that the identified issues are addressed and validation studies are completed.

## Appendices

Appendix 

**Table 4:** Selected items from the PedsQL™ 3.0 Neurofibromatosis Type 1 Module (Ages 8–12). Adapted from the original instrument developed by JW Varni, Ph.D. (©1998) [12]. The instrument is used with permission from the author and the MAPI Research Trust. The full version is available at: https://eprovide.mapi-trust.org/instruments/pediatric-quality-of-life-inventory-neurofibromatosis-type-1-module (accessed on July 21, 2025).

In the past ONE month, how much of a problem has this been for you					
SKIN ITCH BOTHER (problems with…)	Never	Almost Never	Sometimes	Often	Almost Always
1. My skin itches	0	1	2	3	4
2. My skin itches so much it is hard for me to sleep	0	1	2	3	4
3. My skin hurts	0	1	2	3	4
4. Wearing clothes bothers my skin	0	1	2	3	4
5. My skin bothers me more when it is hot	0	1	2	3	4
6. My skin bothers me more when it is cold	0	1	2	3	4
SKIN SENSATIONS (problems with…)	Never	Almost Never	Sometimes	Often	Almost Always
1. My skin tingles	0	1	2	3	4
2. My skin feels like pins and needles	0	1	2	3	4
3. My skin feels like it is burning	0	1	2	3	4
PAIN (problems with…)	Never	Almost Never	Sometimes	Often	Almost Always
1. I hurt a lot	0	1	2	3	4
2. I have pain every day	0	1	2	3	4
3. I have pain so much that I need medicine	0	1	2	3	4
4. I hurt in my stomach	0	1	2	3	4
5. I get headaches	0	1	2	3	4
6. I get backaches	0	1	2	3	4
PAIN IMPACT (problems with…)	Never	Almost Never	Sometimes	Often	Almost Always
1. I have pain so much that I need to lie down	0	1	2	3	4
2. I have so much pain that I have to stop what I am doing	0	1	2	3	4
3. It is hard for me to do things because I might get pain	0	1	2	3	4
4. I miss school when I have pain	0	1	2	3	4
5. It is hard for me to run when I have pain	0	1	2	3	4
6. It is hard to have fun when I have pain	0	1	2	3	4
7. I have trouble moving when I have pain	0	1	2	3	4
8. It is hard to stay standing when I have pain	0	1	2	3	4
9. It is hard for me to take care of myself when I have pain	0	1	2	3	4
10. It is hard for me to do what others can do because I might get pain	0	1	2	3	4
11. It is hard for me to sleep because I have pain	0	1	2	3	4
12. I get tired when I have pain	0	1	2	3	4
13. It is hard for me to sleep without pain medication	0	1	2	3	4
14. It is hard for me to pay attention when I have pain	0	1	2	3	4
15. It is hard for me to do activities with my friends when I have pain	0	1	2	3	4
16. When I am in pain, I try to stay very still	0	1	2	3	4
In the past ONE month, how much of a problem has this been for you …					
PAIN MANAGEMENT (problems with…)	Never	Almost Never	Sometimes	Often	Almost Always
1. It is hard for me to manage my pain	0	1	2	3	4
2. It is hard for me to control my pain	0	1	2	3	4
COGNITIVE FUNCTIONING (problems with…)	Never	Almost Never	Sometimes	Often	Almost Always
1. It is hard for me to pay attention to things	0	1	2	3	4
2. I forget schoolwork that I need to do	0	1	2	3	4
3. I forget things easily	0	1	2	3	4
4. It is hard for me to understand what I read	0	1	2	3	4
5. It is hard for me to pay attention in class	0	1	2	3	4
6. It is hard for me to remember more than one thing at a time	0	1	2	3	4
7. It is hard for me to remember to do things	0	1	2	3	4
8. It is hard for me to remember what I was just thinking	0	1	2	3	4
9. It is hard for me to do math	0	1	2	3	4
10. It is hard for me to remember what I just read	0	1	2	3	4
11. It is hard for me to remember what people tell me	0	1	2	3	4
12. It takes me longer than other kids to get my schoolwork done	0	1	2	3	4
13. It is hard for me to get started on things	0	1	2	3	4
14. It is hard for me to think quickly	0	1	2	3	4
15. It is hard for me to learn new things	0	1	2	3	4
